# The role of community health workers in addressing the global burden of ear disease and hearing loss: a systematic scoping review of the literature

**DOI:** 10.1136/bmjgh-2018-001141

**Published:** 2019-03-01

**Authors:** James O'Donovan, Misha Verkerk, Niall Winters, Shelly Chadha, Mahmood F Bhutta

**Affiliations:** 1 Department of Education, University of Oxford, Oxford, UK; 2 Division of Research and Health Equity, Omni Med, Mukono, Uganda; 3 Department of Otolaryngology, King's College Hospital NHS Foundation Trust, London, UK; 4 WHO Programme for Prevention of Deafness and Hearing Loss, World Health Organization, Geneva, Switzerland; 5 Department of ENT, Brighton and Sussex University Hospitals NHS Trust, Brighton, UK

**Keywords:** Community Health Worker (CHW), hearing, audiology, hearing-loss, ear disease, hearing screening

## Abstract

**Introduction:**

Community health workers (CHWs) have the potential to improve access to ear and hearing services for people across low-income or middle-income countries, remote, underserved, or resource-poor areas of the world. We performed a systematic scoping review to identify evidence on how CHWs are currently deployed in the prevention, screening, diagnosis, treatment and management of ear disease and hearing loss; methods to train and support CHWs in this context; and cost-effectiveness of CHWs.

**Methods:**

We performed a systematic search of the literature from September 1978 to 18 March 2018 from 11 major databases and the grey literature.

**Results:**

We identified 38 original studies that met the inclusion criteria, taking place across South Asia (n=13), Oceania (n=7), North America (n=7), South America (n=6) and Africa (n=5). 23 studies showed CHWs can increase community participation in screening. They can conduct screening using whispered voice tests, noisemakers for neonatal screening, automated audiological tests and otoscopy. Eight studies focused specifically on the evaluation of programmes to train CHWs, and three provided a general programme description. Three studies documented a role of CHWs in the treatment of ear disease or hearing loss, such as performing ear washouts, instillation of topical antibiotics or fitting of hearing aids. Only one study provided an indepth cost-utility analysis regarding the use of CHWs to conduct hearing screening, and no studies commented on the role of CHWs in the prevention of hearing loss.

**Conclusion:**

CHWs have been employed in diverse ways to address the global burden of ear disease and hearing loss. Future research needs to explore the role of CHWs in preventative strategies, identify optimum methods to train and support CHWs, and explore their cost-effectiveness.

**Key questions**

**What is already known?**

- The global burden of disabling hearing loss is unequally distributed, with 80% of those affected living in low-income or middle-income countries (LMICs).
- To address the shortage of specialist ear care professionals in LMICs and resource-poor areas of the world, community health workers (CHWs) have been proposed as one strategy to fill the gap in human resources for health.

**What are the new findings?**

- This systematic scoping review is the first to try and identify a role of CHWs to improve access to ear and hearing services for people across LMICs, remote, underserved or resource-poor areas of the world.
- We identified 38 original studies that met the inclusion criteria.

**What do the new findings imply?**

- CHWs could potentially play an important role in improving access to ear and hearing services, including in screening, raising community awareness and delivery of basic treatment.
- Future research needs to explore the role of CHWs in preventative strategies, identify optimum methods to train and support CHWs, and explore their cost-effectiveness.

## Introduction

Hearing loss has an enormous global economic impact, estimated at 750 billion international dollars annually, with 63%–73% of these costs incurred in low-income or middle-income countries (LMICs).^1^ Yet, given the multitude of other competing issues, hearing loss is rarely acknowledged,^2 3^ let alone prioritised in resource-limited settings, and as such has been termed ‘the invisible disability’.^4^ At an individual level, hearing loss in childhood can cause oral language and communication impairment, leading to adverse effects in educational attainment and behaviour.^5 6^ In adults or the elderly, hearing loss is associated with depression, dementia and social isolation.^7–9^

Importantly, the global burden of disabling hearing loss is unequally distributed.^10^ Of the 6.1% of the global population affected,^11^ the prevalence of child and adult hearing impairment is substantially higher in LMICs, most of which lack the human or other resources to prevent, diagnose or treat such disease.^12 13^ A WHO report from 2013 revealed that 64% of participating countries from the African region had fewer than one ear, nose and throat (ENT) surgical specialist available per million people, compared with 12 in some high-income countries.^14^ Similarly, 88% of high-income countries reported availability of more than one audiologist per million population, compared with only 5% in low-income countries.^14^ Furthermore, it is important to note that even within countries, ENT surgeons and audiologists are unequally distributed.^15^ Training highly skilled health workers, such as audiologists or surgeons, is expensive, time-consuming, and reinforces a hospital-based model of care that may fail to reach disadvantaged or remote populations. One proposed solution for the recognition and treatment of ear disease and hearing loss is task shifting to cadres of health workers with less specialised training, such as community health workers (CHWs).^3 16 17^

In the broadest sense, CHW is an umbrella term for laypeople working within their own community in a health promotion, prevention and delivery role^18^; however, the nomenclature used to describe CHWs is wide-ranging, and their exact roles, responsibilities, recruitment, remuneration and training vary from country to country.^19^ CHWs have been successful in providing care for infectious diseases, and for maternal and child health,^20^ and this approach could be emulated for ear and hearing health.^1^ This concept is not new; basic ear care workers have been active in rural indigenous communities in Australia and Canada since the 1980s.^21 22^

In this scoping review we mapped the existing literature for evidence on the role CHWs may play in addressing the global burden of ear disease and hearing loss. Specifically we sought evidence to evaluate four predetermined questions:

1. How are CHWs currently deployed in the prevention, screening and diagnosis of ear disease and hearing loss?
2. How are CHWs deployed in the treatment and management of ear disease and hearing loss?
3. What methods exist to train and support CHWs in ear disease and hearing loss? What are the contents, duration and outcomes of such training?
4. What is the cost-effectiveness of using CHWs in ear disease and hearing loss?

## Methods

### Nature of review

We conducted a scoping review on the role of CHWs in ear disease and hearing loss to identify existing evidence and gaps in the literature. A scoping review is defined as that which addresses an exploratory research question through mapping key concepts, types of evidence and gaps in research by systematically searching, selecting and synthesising knowledge in a field.^23^ Hence, scoping reviews provide a broad overview and organisation of existing knowledge, as opposed to the narrow synthesis of a predefined research question typical of a systematic review.^24–26^ They also place less emphasis on the critical appraisal of the included evidence compared with a traditional systematic review.^26^

A scoping literature review was chosen for this study since it enabled us to review a broad body of literature and map the current ways CHWs are trained and deployed to deal with ear disease and hearing loss across a variety of different geographical contexts.

### Search strategy and study selection

A search of the Cochrane Library, the Campbell Collaboration, the International Prospective Register of Systematic Reviews (PROSPERO) and grey literature identified no existing or scheduled reviews on this topic.

We designed an exhaustive and sensitive search strategy through developing terms for ‘community health workers’, ‘hearing loss’ and ‘ear disease’. There were combined using the AND operator in a master search string (see table 1, online supplementary material). Where appropriate, Medical Subject Headings (MeSH) terms were exploded to relevant subheadings, and synonyms searched for each key term, along with wildcards and truncation for free-text words.

10.1136/bmjgh-2018-001141.supp1 Supplementary data

**Table 1:** Roles of CHWs in ear and hearing disease that have been reported in the literature or have potential (as indicated by an asterisk)

Training level	Basic	Intermediate	Advanced
Screening and diagnosis.	Encouraging community participation.	Taking a clinical history.* Clinical tests of hearing (whispered voice test, noisemakers for neonatal screening). Automated audiometry (eg, PTA, OAE, ABR). Otoscopy (store and send). Otoscopy (diagnostic).*	Diagnostic audiometry (eg, PTA, paediatric audiological tests).
Treatment.	Raising awareness.* Preventative measures.*	Ear washout. Antibiotic therapy. Dry mopping of the ear.*	Fitting and maintenance of hearing aids.

ABR, auditory brainstem response; CHW, community health worker; OAE, otoacoustic emissions; PTA, pure tone audiometry.

In this study (and consistent with agreed definitions),^27 28^ we defined CHWs as health workers who are members of the communities where they work, but without formal professional or paraprofessional certificated tertiary education. They should work in the community (rather than a health facility), belong to the formal health system (managed by the government or non-governmental organisation) and perform tasks related to healthcare delivery.^19 20^

We searched the following databases for studies published between 12 September 1978 (the date of the Alma Ata Declaration, which declared CHWs as central to primary healthcare)^29^ and 18 March 2018: Medline; Embase, Allied and Complementary Medicine Database and Global Health via Ovid; Cumulative Index to Nursing and Allied Health Literature via Ebsco; PsycINFO; Web of Science; Scopus; Applied Social Sciences Index and Abstracts via ProQuest; British Education Index; and Education Resources Information Center (the full search strategy for each database is listed in online supplementary table 1). We included additional non-peer-reviewed literature identified through the e-theses online service (ETHoS), Google Scholar, and websites of research institutions, charities, relevant government departments and international agencies involved in ear and hearing care. We also conducted a manual search of grey literature databases (see online supplementary table 2). Finally, we searched the reference lists of all relevant papers identified, using snowball sampling. No restrictions were placed on language.

Regarding inclusion and exclusion criteria, studies were included if:

1. The primary participants of the study were CHWs (as per the definition outlined above).
2. The primary aim of the study was to describe or evaluate the role of CHWs in the prevention, screening, diagnosis, treatment and/or management of ear disease and/or hearing loss.

Studies were excluded if:

1. The primary focus of the study was on health workers other than CHWs (eg, doctors, medical students, nurses or allied healthcare professionals, such as midwives).
2. The study did not contain sufficient information to extract meaningful information regarding the role of CHWs in ear disease and/or hearing loss. For example, studies which focused on describing the roles of CHWs in managing a broad range of diseases but only mentioned their role in ear disease or hearing loss in passing were excluded.
3. The study was not a primary research or descriptive study relevant to addressing the four predetermined research questions. We excluded letters, commentaries, opinion pieces, study protocols, policy briefings, training needs assessments and conference abstracts.

Identified papers were exported into EndNote V.7.1 and duplicates were removed. Titles and abstracts were screened by two authors (JOD and MV), and the full text of potentially relevant papers retrieved and reviewed. Data from the full text were extracted and entered into a Microsoft Excel data charting form. Each reviewer had a copy of the inclusion and exclusion criteria to hand when completing this process. Where the reviewer felt the study should be excluded, they noted this in the data charting form and provided a reason for exclusion. Disagreement between reviewers was resolved via discussion and by referring to the inclusion and exclusion criteria. Where needed, we contacted the authors of individual studies for further information.

A review protocol was not published and the study was not registered with PROSPERO, as these mechanisms are not applied to scoping reviews.^23 24^

### Data extraction

JOD extracted and tabulated relevant data from each included study, including the study author, title, date of publication, country and region in which the study took place, CHW name and cadre description, the number of CHWs involved, the primary aims and outcomes of the programme, whether or not government or CHWs were involved in programme planning or design, financial data, details of training, the focus theme of the paper, and whether eHealth or telemedicine was used. MV checked data extraction for accuracy. At all stages, disagreements between the review authors were resolved via discussion.

## Results

### Search results

**Figure 1:**
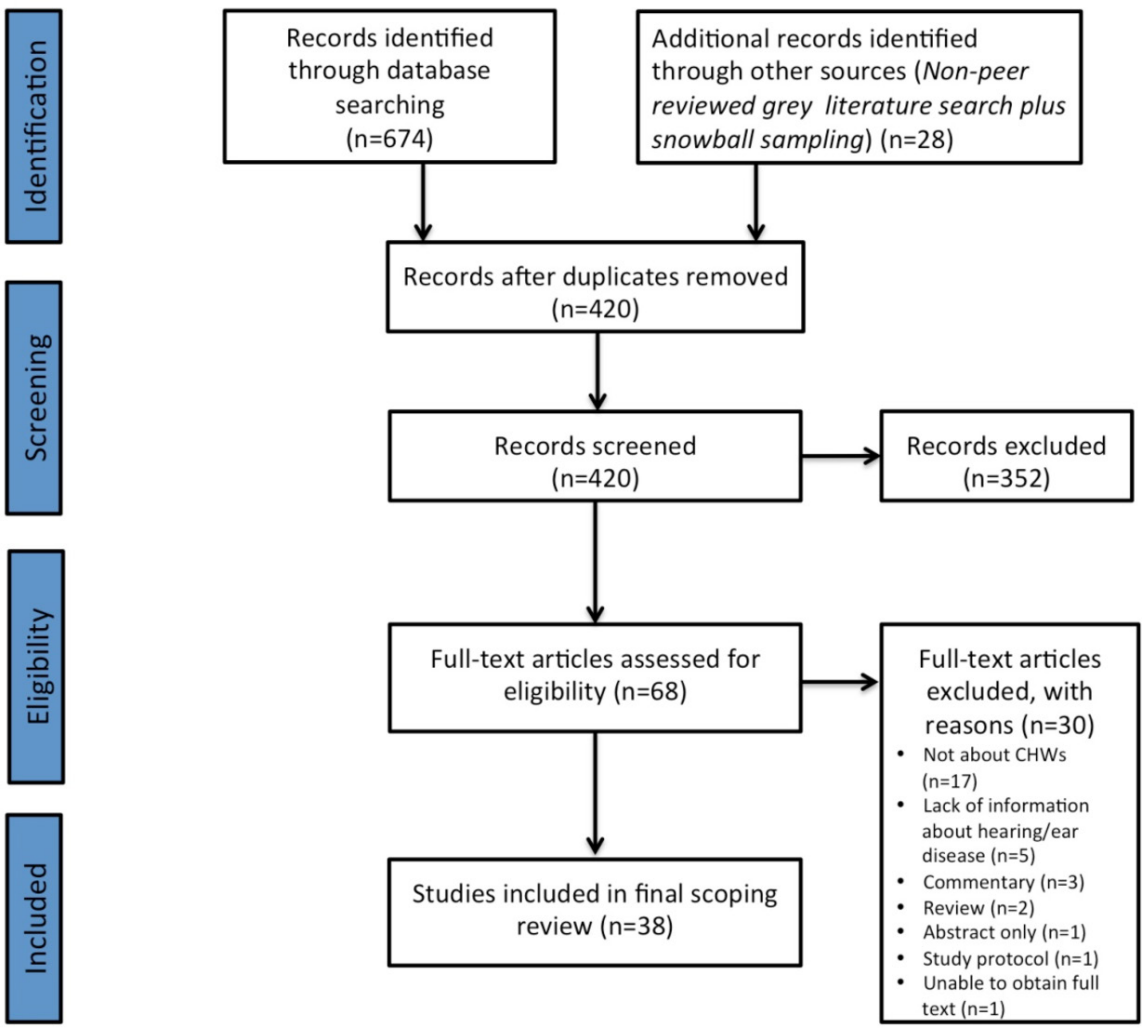
PRISMA diagram. The PRISMA diagram details our search and selection process applied during the scoping review. CHWs, community health workers; PRISMA, Preferred Reporting Items for Systematic Reviews and Meta-Analyses.

The initial search yielded 674 articles, which reduced to 420 after removal of duplicates (see online supplementary table 3). After screening of abstracts, 356 studies were excluded and after full-text review a further 30, leaving 38 studies that met the inclusion criteria.^21 22 30–65^ Further details are in the Preferred Reporting Items for Systematic Reviews and Meta-Analyses flow chart (figure 1).

### Characteristics of included studies

Across the studies 15 different terms were used to define CHWs, with significant variations in cadre roles, responsibilities and status. Some CHWs, such as the Siutilirijiit and Aaniasiurtiapiit in Canada, were full-time workers focused solely on the provision of ear care.^35 37^ In other studies CHWs had additional responsibilities, such as providing maternal and child healthcare.^52 57^

The majority of studies have been published in the past decade (since 2008, n=30), with no relevant studies published before 1980. Studies took place in South Asia (n=13),^30 36 43–45 48 55–59 61 65^ Oceania (n=7),^21 38 39 41 42 62 63^ North America (n=7),^22 35 37 47 49 50 60^ South America (n=6)^31–34 40 64^ or Africa (n=5).^46 51–54^

Full details of CHW cadre descriptions and study characteristics can be found in online supplementary table 4.

### Prevention, screening and diagnosis of ear disease and hearing loss by CHWs

We identified 23 studies which primarily focused on the role of CHWs in screening for ear disease and hearing loss.^21 30 31 36 42 43 45–47 49–51 53–56 58 59 61–64 66^ There were no studies on the role of CHWs in disease prevention.

In the most basic terms, CHWs have been used to facilitate community attendance at hearing screening camps run by experts.^44 61^ In India, Emerson *et al* 44 reported that CHWs mobilised 2600 patients from the community to attend 20 hearing screening camps held in the Vellore District of Tamil Nadu between 2009 and 2011. In Nepal, Shrestha *et al* 61 reported that using CHWs to mobilise rural community members resulted in a 20% increase in attendance at hearing camps over an 18-month period.^61^

CHWs have also been evaluated for their own potential for undertaking screening, to identify either hearing loss^30 31 36 43 46 48 50 53–56 58 64^ or other ear disease.^21 38 42 44 45 49 59 61–63^

Alvarenga *et al* 31 used a locally devised questionnaire administered by 66 CHWs in Brazil to screen for childhood hearing loss. They screened 304 children; however, the sensitivity of the questionnaire was poor: only 1 of 69 children with conductive hearing loss and 4 of 6 with sensorineural hearing loss were identified (no data were provided on the severity of hearing loss identified). Wagner *et al* 64 gave a similar questionnaire to a cadre of CHWs to screen for infant hearing loss in Brazil. This required monthly administration of the questionnaire for 6 months, and was only used with parents of infants who had already passed newborn hearing assessment. Only 15% (6/41) of CHWs completed the study, with attrition over the duration of the study. Many CHWs cited high existing workloads as the reason for non-completion,^64^ but additional factors may have been that the screened population was at low risk of disease, that the questionnaire itself had not been validated, and fatigue set in given the recommendation to administer the questionnaire every month to the same participants.

The whispered voice test is established as a valid clinical screening test for hearing loss in adults and older children, but most studies have evaluated its administration by experts only.^67^ In a broad screen of disability in 150 frail and elderly adults, Jotheeswaran *et al* 48 evaluated the whisper test administered by CHWs in India, compared with that by physicians with over 10 years of primary care experience.^48^ Of those screened, 104 failed assessment by CHWs, with 71% concordance with the physicians. It is important to note that the assessment by both the CHWs and physicians did not include audiometry, and so the true validity of this approach remains unclear, nor is it known if this approach may be valid in other populations.

Yousuf Hussein *et al* 46 conducted a study in South Africa exploring CHWs’ use of the automated pure tone audiometry (PTA) screening mobile phone application hearScreen which has previously been validated for accuracy.^68^ In this study 12% of adults and 6.5% of children from the community were referred for further assessment, and CHWs reported the method to be acceptable and time-efficient.^46^ PTA conducted by CHWs was mentioned in several studies; however, details of how the CHW were trained to conduct complex audiological testing were not provided.^42 43 62^ Elliott *et al* 42 reported that 157 of 442 (35%) Australian Aboriginal patients screened by PTA were referred to a specialist, but did not report the validity of the PTA performed by CHWs. No other study reported on the validity of CHW-conducted PTA.

CHWs have also been trained in play audiometry. Berg *et al* 36 described a 2-week training course for CHWs in Bangladesh,^36^ followed by screening with play audiometry of 4003 children aged 2–9 years old. However, they found that 66% of the younger children (aged 2–5) were untestable, which perhaps reflects the complexity and special skillset required to test hearing in young children. No study reported on the validity of CHW-conducted play audiometry.

For neonatal hearing loss, Ramesh *et al* 55 described screening by CHWs in rural India using a locally made noisemaker consisting of a wooden cylinder filled with plastic and metal beads. Of the 425 neonates screened, 20 were purposively selected with significant hearing loss (requiring aiding). When performed at a 70–80 dB(A) stimulus, this method was found to have 100% sensitivity and 95%–97% specificity, compared with the gold-standard otoacoustic emissions (OAE) and auditory brainstem response (ABR) testing by an audiologist.

Automated neonatal screening tests can also be performed by CHWs. Akilan^30^ demonstrated that CHWs in India could undertake OAE testing following a 2-week training programme, but noted that the time taken to conduct screening was between 30 and 120 min, which is much longer than the 3–8 min to perform OAE testing reported in high-resource settings.^69 70^ Background noise was also noted to be an issue for screening in the community.^30^ Similarly, Olusanya *et al* 54 reported an 88% success rate of CHWs in Nigeria performing automated OAEs and ABR testing in clinic. But here they noted a significant loss to follow-up in those testing positive, which they surmised to be due to sociocultural factors, including stigma associated with hearing loss.

Several studies have shown that CHWs are able to capture images using otoendoscopy to forward for expert assessment.^38 41 42 45 49^ Kokesh *et al* 49 described capture of postoperative images following tympanostomy tube placement by CHWs in rural Alaska. Remote evaluation of these images by an ENT specialist deemed that 79% of the images were ‘adequate’ for diagnosis. Similarly, Elliot *et al* found that 90% of otoendoscopic image captures by CHWs screening remote indigenous children in Australia (aged 0–16) were deemed ‘excellent’ by a remote ENT specialist.^42^

### Treatment and management of ear disease and hearing loss by CHWs

Three studies described a role of CHWs in the treatment of ear disease and hearing loss.^22 38 43^ In a clinical trial, Couzos *et al* 38 described twice-daily administration of ear washouts and ototopical antibiotic therapy by CHWs for 9–10 days to Australian Aboriginal children with chronic suppurative otitis media. Ear washout and ciprofloxacin eardrops resulted in a 76% cure rate for resolution of otorrhoea at 2 weeks, which compares with success rates for similar treatment regimens in a hospital setting.^71^ Despite such an intensive treatment regimen, over 76% of children (111/147) completed the full treatment course, which may reflect the engagement of the community in this trial.^38^

Emerson *et al* 43 reported that after 6 weeks of targeted training, CHWs in India were able to fit and programme hearing aids, as well as provide counselling to community members who needed to use them. This resulted in a very high acceptance rate of 98% and reduced feelings of shyness among community members wearing a hearing aid.^43^ Specifically trained CHWs have also fitted hearing aids in remote regions of Canada.^22^

### Methods for training and support of CHWs

In eight studies, the primary focus was in evaluating the delivery of CHW training programmes.^32–34 40 41 52 60 65^ There was a great deal of heterogeneity between studies regarding content, duration and method of delivery, making direct comparison difficult.

Some training programmes, such as that by Eikelboom *et al* 41, provided indepth coverage of topics including a theoretical background on the anatomy and physiology of the ear, and an outline of the causes, prevention and treatment of common ear diseases. Other training programmes were focused on one key area, such as diagnostic otoscopy skills.^65^ Five studies referred to the use of the WHO Primary Ear Care Training Manuals as the educational resource,^32–34 40 52^ whereas the remainder did not detail the training content or how it was developed.

Training was delivered mainly through inperson workshops or classes, although four studies exploited digital technologies.^33 34 40 41^ For example, Araújo *et al* 34 used a computer-based ‘Cybertutor’ platform to train 24 CHWs in Brazil.

Training duration was not commonly reported, but where it was, it was often brief, ranging between 3 and 8 hours, in a one-off training session.^33 52^ Interestingly, Araújo *et al* 34 noted that after initial training, assessment scores of CHWs improved, but were significantly worse 15 months later. No studies reported ongoing training of CHWs.

The effectiveness of training was commonly evaluated through the use of pretraining and post-training knowledge assessments^32–34^; however, some studies also evaluated training effectiveness through change in practice. For example, in the study by Mulwafu *et al* 52 in Malawi, health surveillance assistants were asked to identify patients at risk of ear disease and hearing loss to be screened in the community. Of the 1739 patients identified in the community as having potential ear and hearing disorders, they were successfully able to mobilise 860 patients to attend screening camps, of whom 400 had hearing loss. Sanchéz *et al* 60 chose not to evaluate training effectiveness through pretraining and post-training knowledge assessments, and rather focused their evaluation on qualitative assessments of improvements in confidence and the perceived ability of CHWs to provide family support and make referrals for specialist input.

### Cost-effectiveness of CHWs in delivering ear and hearing care

There was a paucity of studies discussing the financial implications of training and using CHWs to provide ear and hearing care. Only Ramkumar *et al* 57 provided sufficient analysis to conclude that using CHWs and tele-ABR to conduct community screening for childhood hearing loss was cost-effective. They compared conducting telediagnostic ABR in a mobile van using satellite connectivity with a local centre using broadband internet in a rural location. According to the authors, the difference in cost between using satellite connectivity in a mobile van versus broadband internet was ‘$1.14 per child screened, $80 per child followed-up and $304 per child identified’.^57^

Several other studies provided basic costs of interventions or programmes, but no cost-benefit or cost-effectiveness analyses. For example, Yousuf Hussein *et al* 46 discussed the costs of the mobile-based hearScreen application, yet failed to provide details on the costs of ongoing technical support and long-term maintenance. Similarly, Olusanya *et al* 53 estimated the cost of infant screening in Nigeria was ‘less than $8 USD’ per child, but did not provide details on how this was calculated.

Akilan *et al* 30, in their evaluation of neonatal hearing screen in impoverished communities in South India, found that 73 of 82 (89%) mothers were willing to pay something for their children to be screened, but only 20 of 82 (24%) could afford the full costs.

Full details of studies are in online supplementary table 4.

## Discussion

This review has identified a number of studies on the use of CHWs in screening, diagnosis and treatment of ear and hearing disease; however, few demonstrated robust outcomes or long-term results. Nevertheless, these studies show that CHWs can be used for delivery of such care, and based on both the evidence presented here and evidence from other contexts (detailed below), we propose a model for incorporation of CHWs into programmes for tackling the burden of ear and hearing disease (see table 1).

At a basic level, the evidence suggests that CHWs can successfully raise community awareness of ear disease and hearing loss, and promote participation in screening programmes. It is likely this requires low-intensity training of CHWs, and that such CHWs could also be deployed to promote preventative measures, such as encouraging vaccination or smoking cessation to reduce risk of otitis media or limitation of noise exposure to prevent noise-induced hearing loss. CHWs have been successfully used in tobacco control measures and vaccination programmes in other contexts.^72 73^

After an intermediate level of training, CHWs could perform clinical examination to screen for disease, including use of otoscopy, whispered voice tests for evaluating hearing in older children and adults, and noisemakers for screening for hearing loss in babies. They could also perform automated tests of hearing, including PTA, OAE and ABR. No studies have evaluated the ability of CHWs to take a clinical history of ear and hearing disease, nor tools to help enable that.

In terms of treatment, Couzos *et al* 38 used CHWs to perform ear washouts and instil antibiotic drops; however, we identified a paucity of studies assessing the role of CHWs in administering treatment. Given appropriate ethical clearance and safety considerations, this is an avenue that should be evaluated in the future. Given the severe shortage of trained ear specialists in LMIC settings, exploring ways to train CHWs to provide simple, low-risk treatment options (such as dry mopping or administering eardrops) may help free up time for more complex disorders to be managed by specialist ear care professionals.

Our review has also identified reports of CHWs successfully performing PTA and fitting and maintenance of hearing aids. This is presumably after a prolonged period of expert tuition; however, exact training details were not provided. It is noteworthy that Berg *et al* 36 reported little success with paediatric audiological testing after training CHWs for 2 weeks, and perhaps a prolonged period of training could have led to greater success here. However, due to the lack of details regarding specific training strategies and outcomes, it is difficult to pinpoint areas for improvement.

### Enabling CHWs: barriers and opportunities

In reality, the role that individual cohorts of CHWs can play in ear and hearing disease will be contingent on many factors. Although there is variation in training and capacity of CHWs across countries, there is no one-size-fits-all approach for CHW programmes. Any programme should be adapted to suit the needs of local contexts. Some countries or regions do not have CHW availability, and where they do may not recognise ear and hearing disease as a priority.^74^ Where CHWs exist, they may have limited time to devote to ear and hearing disease due to competing priorities, whereas others may have more time or be dedicated ear care workers. Some will have little background knowledge or training in healthcare, others may be well versed in such matters and so more readily able to take on roles. Some may have access only to online or written training resources, others to expert face-to-face tuition. Some will have access to specialist equipment, others will not. This has resulted in a fragmented landscape of CHW programmes, with varying levels of resources and support.^75^

It is also important to recognise barriers to community participation. For example Gupta *et al* 45 noted that many community members in northern India did not understand the need for hearing screening and several refused participation due to fear. In Nigeria, Olusanya *et al* 54﻿ noted delay in follow-up of infants identified as at risk of hearing loss, perhaps because community members did not view such disease as life-threatening or urgent, or because of associated stigma. In impoverished regions of eastern India, Ramkumar *et al* 57 reported that many at-risk children were not brought for follow-up testing of hearing due to prohibitive costs of travel and potential loss of earnings. Mulwafu *et al* 52 found that almost 50% of villagers in Malawi identified as having potential ear and hearing disorders did not attend a hearing screening camp because it clashed with a fertiliser handout. Taking a human-centred design approach towards community-based hearing health programmes may therefore help to reduce such challenges in the future.^76^ Giacomin^77^ defines human-centred design as the use of techniques ‘which communicate, interact, empathize and stimulate the people involved, obtaining an understanding of their needs, desires and experiences which often transcends that which the people themselves actually realized’. Such approaches have been used successfully in other health conditions such as cancer,^78^ HIV and tuberculosis^79^ to help improve patients’ overall experience of care, tailor approaches in a contextually appropriate manner and improve the delivery of health services.

An additional barrier is the availability of incountry expert services. Screening programmes may be futile if treatment of disease is not available,^80^ and in most LMICs the availability of hearing aids, audiologists, ENT surgeons, or speech and language therapists is sparse or non-existent.^74^ It is important that the level of clinical care provided by CHWs is commensurate with their standard of training and skill. There should be robust systems for onward specialist referral in complex clinical cases.^81^ Clear information on the specific care provided by CHWs may help protect CHWs from unrealistic expectations within local communities. As highlighted by the WHO,^18^ CHWs cannot be seen as a panacea to solving the burden of global ear disease, and governments must continue to invest in audiology, speech and language, and ENT services, which need to be developed in parallel with the training of CHWs. Recent thinking has shifted towards ‘how CHWs can support health systems beyond vertical interventions’.^82^ CHWs should be seen as one piece of a complex puzzle, which requires investment at all levels from primary through to tertiary care. It also requires that CHWs are paid, receive ongoing training and are supported for long-term retention by the health systems within which they are located.^82^

### Review limitations

It is important to note that CHWs work within complex health systems, which are significantly variable between individual countries. This means the generalisability of findings across studies to different contexts is difficult. There was a high concentration of studies in South America, Oceania and South Asia. We would encourage further studies across a wider variety of geographical locations.

Second, although we have tried to create a search strategy using as many possible relevant terms and using the most widely accepted definitions, there is no fixed definition of a CHW, and so some exclusions may be contested.

Finally, given the nature of scoping review, a critical appraisal of studies was not undertaken. This represents a limitation since we are unable to comment on the quality of the included studies.

### Future needs

There are several opportunities to promote CHW participation in ear and hearing care.

The value of existing training resources is not known. The WHO Primary Ear Care Training Manuals were the single most commonly cited training material, although many studies did not provide details of resources used to train CHWs. Most studies used pretraining and post-training multiple-choice questions as their outcome measure of learning.^32 33 40 41^ There is a risk that such measures equate exposure to information as a proxy for education,^83^ rather than measures such as the ability of CHWs to diagnose and treat ear disease in a real-world environment. Future studies on training may benefit from participation of CHWs in the development of learning resources, evaluation of practical outcomes rather than knowledge-based outcomes, and evaluation of long-term rather than short-term educational outcomes.^84^ Digital or mobile technologies could enhance delivery of training and provision of ongoing expert support,^83 85^ which have shown promise in other areas of CHW training.^86^

Additional factors that may enable CHWs is the development of relevant low-cost technologies, including otoscopes, noisemakers, audiometers, hearing aids and training aids. The availability of tools for automated mobile phone-based hearing screening is a promising development.^46 58 87 88^

Finally, more work is needed to explore the cost-effectiveness of CHWs in ear and hearing care, factoring costs of recruitment, training and ongoing support, and models for reimbursement.^89^

## Conclusion

CHWs have demonstrated the potential to address the gap in ear and hearing services for the screening and treatment of disease. This approach offers promise both in terms of strengthening local and national health systems and carries positive implications for individuals and society more broadly. However much of the existing research has not explored their role in preventative strategies, optimum methods to train and provide ongoing support for CHWs, nor identified if such models are sustainable and cost-effective. Future studies should address these knowledge gaps with high-quality evidence, as well as explore additional barriers to CHW participation, including the provision of affordable technologies for diagnosis and treatment, and community engagement.
