# Supplementary material for: The role of community health workers in addressing the global burden of ear disease and hearing loss: a systematic scoping review of the literature
Source: BMJ Glob Health. 2019 Mar 1;4(2):e001141. doi: 10.1136/bmjgh-2018-001141 (PMC6407559; doi:10.1136/bmjgh-2018-001141)
Supplement: Supplementary data [file bmjgh-2018-001141supp001.pdf]

Supplementary Material

Database	Search Terms
**Medline, CINAHL, ERIC, BEI**	((“community health*” N3 (worker* or volunteer or aide* or practitioner* or supporter)) or (“village health*” N3 (worker* or team* or guide*)) or “lady health worker*” or “lady health visitor*” or (front?line primary health?care) or behvarz or brigadista or manzaneras or “rural health assistant*” or gramsakhi or “lay health worker*” or “trained birth assistant*” or “accredited social health activist*” or “adherence support worker*” or (community N4 “treatment support*”) or (community* N3 (distributor* or “care worker*”)) or “health extension worker*” or “promoteras” or “lay health worker” or “lay counselor*” or “maternal health worker*” or “peer educator*” or “bare?foot doctor*” or “shasthy? shebika*” or “shasthy? kormi*” or “front line primary health?care worker” or “health activist*”)) AND ((deafness OR "hearing?loss" OR "hard of hearing" OR "ear" OR "audiology" OR "otology" OR "hearing"))
**AMED (Allied and Complementary Medicine) 1985 to March 2018**<br><br>**Embase 1974 to 2018 Week 10**<br><br>**Global Health 1973 to 2018 Week 10**<br><br>**PsychInfo 1987 to March Week 3 2018**	1. (community adj1 health* adj3 (worker* or volunteer or aide* or practitioner* or supporter)).af.<br>2. (village adj1 health* adj3 (worker* or team* or guide*)).af.<br>3. lady health worker.af.<br>4. (lady health visitor* or frontline primary healthcare).af.<br>5. (behvarz or brigadista or manzaneras or rural health assistant* or lay health worker or lay counselor or gramsakhi or lay health worker* or trained birth assistant* or accredited social health activist* or adherence support worker*).af.<br>6. (community adj3 treatment support*).af.<br>7. (community adj3 distributor*).af.<br>8. (health extension worker* or lay counsellor* or promoteras or maternal health worker* or peer educator* or barefoot doctor* or shasthy? shebika* or shasthy? kormi* or front line primary healthcare worker or health activist*).af.<br>9. (deafness or hearing?loss or hard of hearing or ear or audiology or otology or hearing).af.<br>10. 1 or 2 or 3 or 4 or 5 or 6 or 7 or 8<br>11. 9 and 10
**Web of Science**	**TOPIC:** (((community health* NEAR/3 (worker* or volunteer or aide* or practitioner* or supporter)) or (village health* NEAR/3 (worker* or team* or guide*)) or lady health worker* or lady health visitor* or (front?line primary

	health?care) or behvarz or brigadista or manzaneras or rural health assistant* or gramsakhi or lay health worker* or trained birth assistant* or accredited social health activist* or adherence support worker* or (community NEAR/3 treatment support*) or (community*NEAR/3 (distributor* or care worker*)) or health extension worker* or lay counselor* or lay health worker or maternal health worker* or peer educator* or bare?foot doctor* or shasthy? shebika* or shasthy? kormi* or front line primary health?care worker or health activist*)) *AND* **TOPIC:** (deafness OR "hearing?loss" OR "hard of hearing" OR "ear" OR "audiology" OR "otology" OR "hearing")<br>**Timespan:** 1978-2017. **Indexes:** SCI-EXPANDED, SSCI, A&HCI, CPCI-S, CPCI-SSH, BKCI-S, BKCI-SSH, ESCI, CCR-EXPANDED, IC.
**SCOPUS**	*TITLE-ABS-KEY ((* "community health*" *PRE/3 (* worker* *OR* volunteer *OR* aide* *OR* practitioner* *OR* supporter *)) OR (* "village health*" *PRE/3 (* worker* *OR* team* *OR* guide* *)) OR* "lady health worker*" *OR* "lady health visitor*" *OR (* front?line *AND* primary *AND* health?care *) OR* behvarz *OR* brigadista *OR* manzaneras *OR* "rural health assistant*" *OR* gramsakhi *OR* "lay health worker*" *OR* "trained birth assistant*" *OR* "accredited social health activist*" *OR* "adherence support worker*" *OR (* community *PRE/3* "treatment support*" *) OR (* community* *PRE/3 (* distributor* *OR* "care worker*" *)) OR* "health extension worker*" *OR* "lay counsellor*" *OR "lay health worker" OR* "maternal health worker*" *OR* "peer educator*" *OR* "bare?foot doctor*" *OR* "shasthy? shebika*" *OR* "shasthy? kormi*" *OR* "front line primary health?care worker" *OR* "health activist*" *) AND (* deafness *OR* "hearing?loss" *OR* "hard of hearing" *OR* "ear" *OR* "audiology" *OR* "otology" *OR* "hearing" *)*
**ASSIA via PROQUEST**	ab(("community health*" N3 (worker* OR volunteer OR aide* OR practitioner* OR supporter)) OR ("village health*" N3 (worker* OR team* OR guide*)) OR "lady health worker*" OR "lady health visitor*" OR (front?line primary health?care) OR behvarz OR brigadista OR manzaneras OR "rural health assistant*" OR gramsakhi OR "lay health worker*" OR "trained birth assistant*" OR "accredited social health activist*" OR "adherence support worker*" or “health extension worker*” or “lay health worker” or “lay counselor*” or “maternal health worker*” or “peer educator*” or “bare?foot doctor*” or “shasthy? shebika*” or “shasthy? kormi*” or “front line primary health?care worker” or “health activist*” or (community N4 “treatment support*”) or (community* N3 (distributor* or “care worker*”))) AND (deafness OR "hearing?loss" OR "hard of hearing" OR "ear" OR "audiology" OR "otology" OR "hearing")

Supplementary Table 1. Database search strategies, including search terms. The full search strategy used for each of the 11 databases that were searched.

Sources
Google Scholar (https://scholar.google.com/)
E-theses online service (https://ethos.bl.uk)
Mobile Active (http:// www.mobileactive.org)
KIT Information Portal (http://labquality.kit-ipp.org/)
WHO publication database (http://www.who.int/publications/en/)
Knowledge for Health website (https://www.k4health.org/)
Open Grey (http://www.opengrey.eu/)
Proquest Dissertations & Theses (http://www.proquest.com/products-services/pqdtglobal.html)
International Child Health Review Collaboration website (http://www.ichrc.org/)
UNICEF website (https://www.unicef.org/)
WHO website (http://www.who.int/en/)
USAID website (https://www.usaid.gov/)
Clinicaltrials.gov website (https://clinicaltrials.gov/)
WHO Clinical Trials Registry Platform (http://www.who.int/ictrp/en/)
CHW Central Website (http://www.chwcentral.org/)
Bill & Melinda Gates Foundation Website (https://www.gatesfoundation.org/)
Medic Mobile Website (https://medicmobile.org/)
One Million Community Health Workers website (http://1millionhealthworkers.org/)
Frontline Health Workers Coalition website (https://www.frontlinehealthworkers.org/)
Hear the World website (https://www.hear-the-world.com/en)
Action on Hearing Loss website (https://www.actiononhearingloss.org.uk/)
Global Foundation for Children with Hearing Loss website (http://www.childrenwithhearingloss.org/)
The American Academy of Otolaryngology and Head and Neck Surgery Global Health section (http://www.entnet.org/content/otolaryngology-global-health)
World Wide Hearing website (http://www.wwhearing.org/)

Supplementary Table 2. Grey literature sources. A table outlining the sources used as part of the search for 'grey literature'.

Database	Number of hits	Page number for search details
Medline	61	1
CINAHL	17	1
ERIC	6	1
BEI	1	1
Embase	60	1
Allied and Complementary Medicine (AMED) via Ovid (1985 to March	0	1
PsychInfo	103	1
Global Health	20	1
Web of Science	281	2
Scopus	63	2
ASSIA via ProQuest	62	2

Supplementary Table 3. Database search results. A table outlining the number of results generated from the search of each individual database.

Authors	Study Title	Year	Country and Region	Name given to CHW	Cadre description	Primary thematic focus of paper	CHW Training content, duration and delivery	Use of eHealth or tele-medicine?	Study outcome(s)	Costs or financial details
Akilan et al.	Perception of 'mothers of beneficiaries' regarding a rural community based hearing screening service	2014	India, South Asia	Village Health Workers (VHWs)	No general cadre description provided	**Screening.**<br><br>This study focused on hearing screening of infants and young children using Oto Acoustic Emissions (OAE) equipment. When OAE failed, telehealth technology with Auditory Brainstem Response (ABR) was used.	**Content:** An introduction to ear and hearing; the need for hearing screening; methods for screening hearing; how to give information; how to collect data; identification of risk factors for hearing loss; provision of counseling<br>**Duration:** Two weeks<br>**Trainer:** Audiologist	Telhealth ABR for those who fail OAE	Mothers in the community accepted hearing screening services delivered by VHWs . The health workers were effective in delivering the services. Pre-school teachers seemed to have played a pivotal role in communicating about the hearing screening program to the mothers. Ultimately, collaborating with a local NGO facilitated acceptance and compliance due to the NGOs strong presence in the community.	Mothers were informed that the hearing test would cost between 400-500 rupees. Of the 83 mothers who took part in the focus discussion groups, twenty reported that they could spend around 500 rupees on a hearing test, whereas five mothers reported they could only spend less than 100 rupees.
Alveranga et al.*	Questionnaire for monitoring auditory and language developme	2013	Brazil, South America	Community Health Agents (CHAs)	No general cadre description was provided	**Screening.**<br><br>This study focused on hearing screening of infants aged 0-	**Content:** General training on infant hearing loss. The questionnaire was adapted from the WHO training materials.	No details	304 children were screened.<br>Four children (1.32%) were diagnosed with sensorineural hearing loss (two unilateral), and 69 (22.7%) with conductive	No details

	nt in the first year of life					1 years old using a questionnaire distributed by CHWs to family members. Follow up was carried out by an audiologist and involved OAE, the Ling Six-Sounds Hearing test and acoustics immittanciometry.<br><br>Infants who had altered hearing on any of the applied tests were referred to the SALT department at the University of Sao Paulo and underwent medical evaluation and supplementation of the audiological evaluation	**Duration:** 8 hours<br>**Trainer:** No details		hearing loss. The monitoring questionnaire showed specificity of 96% and sensitivity of 67%.<br><br>The authors reported that the questionnaire helped to raise families' awareness of the hearing development of their children and encouraged them to ask questions about the causes of hearing loss.	

						with the research of the brainstem auditory evoked potentials (BAEP) and audiometry with visual reinforcement, and if necessary, specific treatment.				
Alveranga et al.	Training proposal for community health agents in hearing health	2008	Brazil, South America	Community Health Agents (CHAs)	CHAs were recruited from two separate areas. The average age was 30 years old, with most having secondary school education. None had previously undergone any training in ear care.	**Training.**<br><br>The aim of this study was to verify the effectiveness of a training programme for CHAs regarding the hearing health of children.	**Content:** CHAs were taught about normal and impaired hearing; types, prevention and causes of hearing loss; procedures to identify and diagnose the hearing impairment and rehabilitation. Training materials were adapted from the WHO materials. **Duration:** Eight hours of training	No details	A pre- and post-training questionnaire was used to assess knowledge acquisition and retention before and after the training workshop.<br><br>Improvements in scores were demonstrated across both groups and the authors provided isolated quotes to indicate satisfaction from the community and CHWs.	No details

							**Trainer:** No details on trainer or format of training			
Araújo et al.	Community health worker training for infant hearing health: Effectiveness of distance learning	2013	Brazil, South America	Community Health Workers (CHWs)	No general cadre description was provided	**Training.**<br><br>Two groups of CHWs took part in the training. One group had some previous training experience regarding ear and hearing care, the other had none.	**Content:** Causes of hearing loss, diagnosis, treatment, rehabilitation and prevention. Training materials were adapted from WHO training manuals.<br>**Duration:** CHWs received a computer literacy course of 4 hours prior to delivery of the programme, then were trained in two four-hour sessions on how to use the CDROM tool<br>**Trainer:** Delivered via CDRom	Yes – use of CDRom assisted training.	A pre- and post-training questionnaire was used to assess knowledge acquisition and retention. Both groups performed better after receiving the training via CDRom.<br><br>Six months after the distance training program, simulations of everyday situations were used to evaluate the information CHWs had retained and their abilities to potentially apply the knowledge to real life practical situations. The group of CHWs who had previously had experience and training in ear care performed better in this assessment than the group of CHWs who had received no training prior to the intervention.	No details
Araújo et al.	Community health workers training on	2015	Brazil, South America	Community Health Workers (CHWs)	No general cadre description was	**Training.**<br><br>The aim of this study was to	**Content:** hearing loss causes, treatment and prevention.	Yes – use of 'CyberTutor', a web-based	A pre- and post-training questionnaire was used to assess knowledge acquisition and retention.	No details

	infant hearing health: information retention				provided	investigate the information retention of CHWs who received training on infant hearing health.	Training materials were based on adapted information from WHO manuals. **Duration:** One day **Trainer:** Guidance was delivered automatically through the 'cybertutor' programme	education system	It was found that after 15 months after the initial training period the scores of the CHWs had significantly worsened leading authors to call for the need for continuous training opportunities.	
Ayukawa et al.	Being Outside of the Box: Audiology in Northern Québec	2014	Canada, North America	Siutilirijiit	No general cadre description was provided	**General program description.**<br><br>This study provided an outline of the role of Inuit hearing specialists to address the burden of ear disease in Northern Québec.	**Content:** General focus on ear disease and hearing loss addition to specialist topics such as calibration, cerumen management, hearing aid trouble shooting. **Duration:** On-going, in addition to one week training courses where more specialist topics are covered	No details	The authors concluded that working in a remote area, in a culture different than one's own involves special challenges that have been met by the inclusion of Inuit support personnel for decades in Nunavik.<br><br>There is a known problem of professional turnover in remote regions that can seriously affect services.<br><br>Experience has shown that generally local workers stay longer than those brought in from south and that the employment of a full time	No details

							**Trainer:** Trained audiologists		Siutilirijiit has helped to address this.<br><br>The authors also documented challenges with this role, including that when a Siutilirijiit is not based in the same locale as the audiologist, there can be problems with providing supervision.<br><br>They conclude that the relationship between the audiologist and community can be greatly enhanced when they work alongside a Siutilirijit who has an intimate understanding of local cultures.	
Berg et al.	Screening methods for childhood hearing impairment in rural Bangladesh	2006	Bangladesh, South Asia	Community Health Workers (CHWs)	No general cadre description was provided	**Screening.**<br><br>This study focused on hearing screening of children aged 2 to 9 years old using conditioned play audiometry	**Content:** CHWs were trained in conditioned play audiometry (CPA) and performing otoacoustic emissions (OAEs) and tympanometry.<br>**Duration:** Two weeks	No details	4003 children aged 0-6 underwent hearing screening using CPA. CPA was feasible for most children in the 6—9 years<br>age range, but not for the younger children due to shyness and lack of cooperation.<br><br>569 children aged 4-6	No details

						(CPA).<br><br>Children aged 2-5 underwent further screening using otoacoustic emissions (OAEs) and tympanometry.	**Trainer:** Audiologist		received both CPA and OAE/tympanometry. 69% were untestable using CPA but only 8.9% were untestable using OAE and tympanometry.<br><br>The authors suggested that if only one method of testing was available OAE/tympanometry would be better as it reduced the need for diagnostic assessment referrals.	
Billard	The Hearing and Otitis programme: a model of community based ear and hearing care services for inuit of Nunavik	2014	Canada, North America	Siutilirijiit and Aaniasiurtiapiit	**Siutilirijiit** assist the audiologists to communicate with patients or clients. They assist when there are language barriers and play the role of cultural counselor. Siutilirijiit are selected for their	**General programme description.**<br><br>This study provided an outline of the role of Inuit hearing specialists to address the burden of ear disease in Nunavik	**Content:** Information on the Hearing and Otitis Programme, anatomy and physiology of the ear, information on hearing, otoscopy and otitis media, hearing evaluation, hearing loss, hearing screening, counselling on hearing loss and hearing aids, public education	No details	This descriptive study provided an outline of the Hearing and Otitis program established in 1990, its model of service delivery, the role of the different contributors to the program, and the challenges it faces. The authors concluded that the program has been successful and has needed to use innovative approaches to overcome the challenges it faces.<br><br>These challenges included the need to provide on-going training in a systematic manner in a	The authors stated that "one of the major outcomes of this project was that funding from the Ministry of Health and Social Services was given to guarantee continuity of the services", however failed to outline exact financial details and associated costs.

					sense of initiative, organizational skills, ability to communicate verbally in English (or French) and Inuktitut, and for being respected by the community. Many siutilirijiit have had either personal or professional experiences with hearing impairment, and therefore are able to share their own experiences		and prevention of hearing problems. **Duration:** On-going on the job in addition to one week training courses where more specialist topics are covered **Trainer:** Trained audiologists		culturally appropriate way as well as ensuring all children under the age of 3 years old were screened for hearing loss.	

					with the population they serve.<br><br>**Aaniasiurtiapiit** provide on-going services to the hearing impaired as needed. Including cultural and linguistic advice, hearing screening in students, hearing aid user follow-up, hearing aid management and minor repairs					
**Couzos et al.	Effectiveness of ototopical antibiotics for chronic suppurative otitis	2003	Australia, Oceania	Aboriginal Health Workers (AHWs)	No general cadre description was provided	**Treatment.**<br><br>This study focused on screening and treatment of CSOM in Aboriginal	**Content:** Training of AHWs included otoscopy skills, video otoscopy/photography capture, and audiometry.	Yes – a sample of digital images of tympanic membranes (using video otoscopy) were copied	147 Aboriginal children with CSOM were recruited, treated and clinically assessed by AHWs. 111 children completed treatment and CSOM resolved in 69% of cases, with	No details

	media in Aboriginal children: a community -based, multicentre, double-blind randomised controlled trial					Children by AHWs.	**Duration:** No details **Trainer:** Provided by the Commonwealth sponsored Australian Hearing program.	onto a CD-ROM and sent to two otolaryngologists for validation of perforation size as reported by AHWs.	significantly greater resolution in the group receiving ototopical ciprofloxacin.	
**Couzos et al.	'We are not just participants - We are in charge': The NACCHO ear trial and the process for Aboriginal community -controlled health research.	2005	Australia, Oceania	Aboriginal Health Workers (AHWs)	No general cadre description was provided	**General program description.** This study described the methodological issues and principles that underpin community-controlled health research as well as practical application. It encourages the adoption of participatory models in research involving Indigenous populations.	**Content:** Trial protocol was discussed during training, however most AHWs had already undertaken a Federal Government-sponsored hearing health training program **Duration:** One week **Trainer:** No details	No details	This descriptive study outlined the key lessons learnt from a community-controlled reaseach study and concluded that community-driven research is strategic and based on priority needs, power differentials were balanced, a holistic approach was taken, community capacity was enhanced, ethical approval was given by the Aboriginal Human Research Ethics Committees, that sustainability had to be considered and realistic timelines set.	Mentioned that community based research could be costly but no exact cost details provided.

De Melo et al.	Community health agents training on hearing health: Effectiveness of videoconference	2010	Brazil, South America	Community Health Agents (CHAs)	No general cadre description was provided	**Training.**<br><br>The aim of this study was to verify the effectiveness of using videoconferencing to train CHAs on child hearing health.	**Content:** General training program on causes, treatment and prevention of hearing loss. Training materials were adapted from WHO materials and translated into Portuguese **Duration:** 8 hours **Trainer:** A facilitator was present in both groups	Yes – use of videoconferencing. 31 community health agents were physically present in the training program and 19 participated via videoconference	Both groups were assessed using pre- and post-training questionnaire assessments. Both groups demonstrated an improvement in scores, however the group who were physically present performed better in all domains compared to the group who only used videoconferencing. The authors concluded that videoconferencing should not replace traditional training but could be used as a complementary delivery tool.	No details
Eikelboom et al.	A tele-otology course for primary care providers.	2003	Australia, Oceania	Aboriginal Health Workers (AHWs)	No general cadre description was provided	**Training.**<br><br>The aim of this study was to assess the effectiveness of a training program for AHWs regarding ear anatomy,	**Content:** General program focusing on hearing loss causes, treatment and prevention. Split into two parts. Part One – Theoretical background e.g. anatomy, physiology,	Yes – tele-otology. The first part of the training course was designed to be delivered to the trainee by CD-ROM or the Internet. This allows didactic	30 AHWs took part.<br><br>Assessment was conducted using a pre-course/post-course MCQ assessment and participant feedback.<br><br>There was also an evaluation of the quality of images that AHWs	No details

						physiology, hearing, otoscopy and telemedicine	pathology of middle ear disease. Part Two – practical demonstration of otoscopy.<br>**Duration:** Part Two was a one day training course<br>**Trainer:** Part One of the course was delivery by digitial methods e.g. CDRom. Part Two was delivered by clinicians.	material to be delivered to the participants in such a way that they can proceed through it when convenient and at their own pace.	were able to obtain of the tympanic membrane using a video otoscope.<br><br>The authors found that the AHWs were able to obtain images of the tympanic membrane that were of reasonable to good quality after a short period of training. There was an average improvement of about 25% in the multiple-choice test scores, and the feedback regarding the course was extremely positive.	
Elliot et al.	The Feasibility of a Community-Based Mobile Telehealth Screening Service for Aboriginal and Torres Strait Islander Children in Australia.	2010	Australia, Oceania	Aboriginal Health Workers (AHWs)	No general cadre description was provided, however the one AHW involved in this study had "*advanced hearing-health training and*	**Screening.**<br><br>Hearing screening of children aged 0-16 was carried out by an AHW in a mobile van. They had an otoscope, tympanometer and audiometric testing equipment	**Content:** No details<br>**Duration:** No details<br>**Trainer:** No details	Yes – telemedicine. A van fitted with screening equipment and telemedicine capabilities was used to screen Indigenous children aged 0-16 who were assessed by an AHW. Screening data and	Over a period of 6 months in 2009, 442 children were screened. Of the 183 (41%) children who failed ear screening, 59 were reviewed remotely by an ear, nose, and throat surgeon, with 9 children booked for surgery. It is important to note however that although the ENT specialist rated 90% of the images viewed as 'good' or 'excellent', 10% were deemed as being too poor	No details

					extensive previous experience in ear health"	available to them.		videootoscopic images were uploaded to a database and made accessible to specialists via a secure website. For those children who failed an ear screening assessment, tele-otology clinics were conducted remotely by an ENTspecialist, who reviewed cases and provided a diagnosis and treatment plan.	quality to make a decision remotely.	
Emerson et al.	Pilot study to evaluate hearing aid service delivery model and measure benefit using self-report outcome	2013	India, South Asia	Communtiy Health Workers (CHWs)	CHWs were selected from the community and it was preferred if they were educated in science subjects.	**Treatment.**<br><br>A range of children and adults were screened in the field by an ENT specialist using an audiometer.	**Content:** CHWs were trained on the principles of basic hearing health care, which included performing pure tone audiometry (PTA) using a portable	No details	111 people in the community with sensorineural hearing loss were identified at a hearing screening camp and had hearing aids fitted over a period of 6 months. Outcomes were assessed using the Abbreviated Profile of Hearing Aid Benefit (APHAB), a 24-	The hearing health care service was provided free of health care costs to the patients at their doorstep by the CHWs.

	measures using community hearing workers in a developing country.					The study reported the role of CHWs to provide comprehensive hearing aid services in the community.	audiometer, impression taking, and ear mould making, performing hearing aid trial and hearing aid fitting, maintenance of hearing aids, minor repairs of hearing aids, and maintenance of ear moulds. They were also taught to counsel patients regarding hearing aid usage.<br>**Duration:** 6 weeks<br>**Trainer:** No details		item self-assessment inventory in which patients report the amount of trouble they are having with communication or noises in various everyday situations.<br><br>The majority of patients reported improved ability to hear, especially in ease of communication and ability to hear despite background noise.	
Emerson et al.	A Model for provision of ENT Health Care Service at Primary and Secondary Hospital Level in a	2013	India, South Asia	Community Health Workers (CHWs)	CHWs were selected from the community and it was preferred if they were educated in science subjects.	**Screening.**<br><br>CHWs were trained to inform the community about common hearing issues and mobilise them to attend an 'ear camp' where	**Content:** CHWs were trained on safe ear care practices, the harmful effects of prevalent traditional practices, and the treatment options available. They were taught to identify the	No details	CHWs were able to successfully mobilise the community to attend an 'ear camp', where specialists were able to diagnose and treat patients.<br><br>2600 patients were evaluated and treated.	No details

	Developing Country					specialist surgeons would be in attendance.<br><br>Children and adults were screened.<br><br>The study also outlined the more general role CHWs play in the community, such as in hearing aid fitting.	symptoms of ear disease and refer patients to the camps conducted periodically. They were also taught how to conduct a hearing assessment using a portable audiometer for adults and fit hearing aids (semidigital, trimmermodel) in the community.<br>**Duration:** No details<br>**Trainer:** No details			
Gupta et al.	Community triage of otology patients using a store-and-forward telemedicin e device: A feasibility study.	2017	India, South Asia	Communi ty Health Workers (CHWs)	CHWs had to have a basic secondary school education and an aptitude for social work.	**Screening.**<br><br>The paper reported the feasibility of CHWs being trained in carrying out screening for ear-disease in the general popuation using a tele-medicine	**Content:** The training curriculum was based on the basic and intermediate modules of the Primary Ear and Hearing Care program developed by WHO. The curriculum included the	Yes – CHWs used ENTreview, a store-and-forward telemedicine device that integrates a camera-enabled smart phone with an otoscope.	45 screening camps were conducted and 3000 patients were screened.<br><br>54% (1,619) were referred for ENT consultation and 215 patients attended. Nearly 50% (103) of the 215 reporting patients required surgical intervention, and 29 patients underwent surgery.	Mentioned need for sustainable financing and a business model but provided no details on how to achieve this. This particular model was a philanthropic: private partnership between Medtronic and a

						otoscopy device.	following broad categories: anatomy and physiology of the ear, ear diseases, examination of the ear, symptomatic approach to ear diseases, and community ear care. Special emphasis was given to patient handling and eliciting clinical history.<br>**Duration:** 3 months<br>**Trainer:** ENT staff at a local hospital			charity hospital in India.<br><br>The screening was free of charge, and surgeries were subsidized at 40% of usual costs.
Hussein et al.	Smartphone hearing screening in mHealth assisted community-based primary care.	2016	South Africa, Southern Africa	Community Health Workers (CHWs)	CHWs were based in the community, had no previous experience in ear or hearing care and typically looked after	**Screening**.<br><br>Hearing screening was carried out of anyone over the age of 4 years old in the community. The screening was performed using	**Content:** General information on childhood hearing screen and training on use of the mobile 'app'<br>**Duration:** 4 hours<br>**Trainer:** No details	Yes – use of hearing screening app 'hearScreen'	108 children (2–15 years) and 598 adults (16–85 years) were screened. Referral rates for children and adults were 12% and 6.5% respectively.<br><br>CHWs perceptions of the app were generally positive in terms of usability,	Details of the cost of the device and app ($120 for the app), headphones ($35), calibration service ($80) was provided but no in-depth financial analysis

					150-200 households.	automated pure tone testing via the hearScreen app during home visits.			need for services, value to community members and time efficiency.	
Ingram et al.	Addressing Hearing Health Care Disparities among Older Adults in a US-Mexico Border Community.	2016	USA, North America	Community Health Workers (CHWs)	CHWs function as liaisons between the clinic and the community to increase access to services and quality culturally competent care.	**Screening.**<br><br>CHWs conducted interviews with people with hearing loss (*n* = 20) and focus groups with their family/friends (*n* = 27) and with members of the community-at-large (*n* = 47) to find disparities in access to hearing care amongst US-Mexico border communities.	**Content:** No details<br>**Duration:** No details<br>**Trainer:** No details	No details	Individuals experienced depression, sadness, and social isolation, as well as frustration and even anger regarding communication. Family members experienced negative impacts of deteriorating communication, but expressed few coping strategies. There was general agreement across data sources that hearing loss was not routinely addressed within primary care and assistive hearing technology was generally unaffordable. Community members described stigma related to hearing loss and a need for greater access to hearing health care and broader community education.<br><br>The authors concluded that CHWs could provide culturally appropriate	No details

									services to address these issues.	
Jotheeswaran et al.	Identifying common impairments in frail and dependent older people: validation of the COPE assessment for non-specialised health workers in low resource primary health care settings.	2015	India, South Asia	Community Health Workers (CHWs)	CHWs undergo nine or 24 months training focused mainly upon midwifery and maternal and child health. Their role is to supplement that of doctors and other higher trained personnel by promoting preventive and curative health activities in the community	**Screening.**<br><br>Hearing screening of elderly adults was conducted using the whisper voice test which was one part of the Caring for Older People (COPE) assessment tool.	**Content:** General training on identification of hearing loss and how to use the COPE assessment tool<br>**Duration:** No details<br>**Trainer:** Two facilitators currently working in Sub-Health Centres of Corlim Primary Health Centre, Goa, India.	No details	10 CHWs carried out screening on 150 people over the age of 60 in their home.<br><br>CHWs found that 104 adults over the age of 60 had hearing impairment. This figure was validated by a clinician who determined 99 adults had hearing impairment.<br><br>The COPE assessment package was generally perceived as easy to administer, with CHWs reporting that they gained knowledge, experience and confidence through training.<br><br>Some CHWs however reported concerns over the time taken to administer the assessment, and its impact on their current workload.	No details

									There was also no discussion around what follow up was given to the adults who were identified as having hearing impairment.	
Kokesh et al.	Digital images for postsurgical follow-up of tympanostomy tubes in remote Alaska.	2008	USA, North America	Community Health Aides (CHAs)	CHAs provide a broad spectrum of care and form the primary care backbone of the Alaska Tribal Health System, allowing patients to access care within their village.	**Screening.**<br><br>CHAs imaged the ears of 35 children aged between 1-16 following tympanostomy tube placement. The patients were then examined in person by two ENT surgeons.	**Content:** Training on how to use the software<br>**Duration:** No details<br>**Trainer:** No details	Yes- images of the tympanic membrane were captured using a video otoscope and AFHCAN telehealth software. These were then sent to an ENT specialist for review.	79% of images obtained by the CHAs were determined as 'adequate'. The authors concluded that video-otoscopy images of the tympanic membrane are comparable to an in-person examination for assessment and treatment of patients following tympanostomy tubes. Store and-forward telemedicine is an acceptable method of following patients post tympanostomy tube placement.	No details

Marrone et al.	Interventional Audiology to Address Hearing Health Care Disparities: Oyendo Bien Pilot Study.	2017	USA, North America	Promoteras de Salud	Members of the public health workforce who develop links between the healthcare system and the community, acting as cultural brokers and educators.	**Screening.**<br><br>CHWs recruited 10 patients with potential hearing loss in the community for screening. They were then screened in the community by an audiologist using audiometry, case history and otoscopy.	**Content:** Information on hearing loss and educating families about hearing loss. Training had content about managing hearing loss as well as specific training on communication skills when communicating with deaf people and their families.<br>**Duration:** No details<br>**Trainer:** No details	No details	Following the screening process, the CHWs facilitied support groups over a period of 5 weeks for 10 older-adults with hearing loss and their family members/friends. The CHWs taught communication strategies and behavioral change. A post-program analysis revealed a perceived decreased in stigma and improved self-coping strategies. One year after the program seven out of the nine adults with hearing loss had sought hearing related health care.	No details

Mukara et al.	Knowledge and care seeking practices for ear infections among parents of under five children in Kigali, Rwanda: a cross-sectional study	2017	Rwanda, East Africa	Cell coordinators	Each district of 100-150 households is supervised by 2 Community Health Workers (CHW) called cell coordinators, assisted by 1 assistant cell coordinator. No previous training in ear disease.	**Screening.**<br><br>This study evaluated parents' and caregivers' knowledge and care seeking practices for ear infections in children under five in Kigali, Rwanda.	**Content:** No details<br>**Duration:** No details<br>**Trainer:** No details	No details	The authors found that parents were generally knowledgeable about the causes, symptoms, prevention, treatment and consequences of ear infections. 89.1% (722) of respondents said they would seek treatment for their children from CHWs. The authors suggest a potential role for CHWs in delivering basic hearing care, that should be reflected in their training.	No details

Mulwafu et al.	Feasibility and acceptability of training community health workers in ear and hearing care in Malawi: a cluster randomised controlled trial.	2017	Malawi, East Africa	Health Surveillance Assistants (HSAs)	HSAs are employed by the Ministry of Health. Each HAS covers a region with approximately 1000 inhabitants. They track pregnancies, births and deaths using their village health registers, conduct health talks and vaccinations. Each receives 12 weeks of training and has important roles in providing care, promoting community	**Training.**<br><br>This was a cluster randomized controlled trial aimed at assessing the feasibility and acceptability of training community health workers (CHWs) in ear and hearing care, and their ability to identify patients with ear and hearing disorders. Patients identified as having potential ear and hearing disorders then underwent screening using otoscopy, pure tone	**Content:** A general training program on hearing loss. Content was delivered through lectures, posters of ear and hearing disorders, flip charts, demonstrations, practical of voice tests, discussion and group work. Material was based on information from the WHO training materials<br>**Duration:** Three days<br>**Trainer:** One ENT surgeon and two clinical ENT officers	No details	Ten health centers participated which comprised of 5 intervention (29 CHWs) and 5 control (28 CHWs) centres.<br><br>Knowledge assessment involved a pre-test and post-test in addition to the intervention group being given 1 month to identify patients with ear and hearing disorders in their communities. These people were screened for hearing disorders by ENT specialists.<br><br>The average overall correct answers increased from 55% to 68% (95% CI 65 to 71) in the intervention group ($p<0.001$). A total of 1739 patients with potential ear and hearing disorders were identified by CHWs and 860 patients attended the screening camps, of whom 400 had hearing loss (73 patients	Yes. The authors estimated the total cost of the training was $189 per trainee, including trainer's costs ($33), trainee's stipend ($64), training supplies ($61) and travel costs ($31).

					participation in healthcare activities and in promoting disease surveillance services at the community level. Prior to this study, they had not received any training in primary ear health care.	audiometry and transient evoked otoacoustic emissions testing performed by an audiologist.			determined through bilateral fail on otoacoustic emissions, 327 patients through audiometry. The intervention was perceived as feasible and acceptable to implement.<br><br>Treatment was given to those identified as having hearing disorders at the screening camp and where appropriate onward referrals were made.	
Olusanya et al.***	Community-based infant hearing screening for early detection of permanent hearing loss in Lagos, Nigeria: a cross-sectional study.	2008	Nigeria, West Africa	Community Health Workers (CHWs)	No general cadre description provided	**Screening.**<br><br>Infants attending for routine immunisation at four primary health centers were invited to take part in hearing screen.<br><br>The first-stage was screening with transient-	**Content:** An overview of the peripheral auditory pathway and basic screening techniques<br>**Duration:** Two weeks<br>**Trainer:** Principle investigator of the research study	No details	2003 (88%) of 2277 eligible infants attending the four routine immunization clinics were successfully screened between July 2005 and April 2006 at a mean age of 17.7 days, with no parent declining screening. The overall referral rate for diagnostic evaluation was 4.1%. Only 61% (50/82) of those referred returned for evaluation, and 45 of	Estimated that the cost of screening per infant was $8 USD but provided no evidence as to how this figure was estimated.

						evoked otoacoustic emissions and second-stage screening involved automated auditory brainstem response. Those who failed the second screening were referred for diagnostic evaluation within three months.			them were confirmed with permanent congenital and early-onset hearing loss. Additionally, 11 infants who had previously passed the first screening stage were also found to have PCEHL, resulting in a yield of 28 per 1000.	
Olusanya et al.***	Community-based infant hearing screening in a developing country: parental uptake of follow-up services.	2009	Nigeria, West Africa	Community Health Workers (CHWs)	No general cadre description provided	**Screening.**<br><br>This was an in-depth analysis of the study above, aimed at understanding factors associated with follow up after hearing screening.	**Content:** A mixed theoretical and skills based course, including (1) history taking in patient with ear and hearing disorders, (2) ear examination, (3) steps in doing otoscopy, (4) steps in doing voice tests and (5) assessment of hearing in children	No details	The authors found that return rates for second stage screening were poor. 285 out of 2,003 eligible infants were referred after the first-stage screening out of which 148 (51.9%) did not return for the second-stage, while 32 (39.0%) of the 82 infants scheduled for diagnostic evaluation defaulted. Mothers who delivered outside hospitals were	No details

							**Duration:** No details<br>**Trainer:** Community nurse lead		significantly more likely to return for follow-up screening than those who delivered in hospitals (Odds ratio: 1.62; 95% confidence intervals: 0.98 – 2.70; p = 0.062), however no other factors correlated with follow-up compliance for screening and diagnostic services.	

Peever et al.	Successful assault on ear disease: intensive daily treatment by nurses and health workers.	1980	Australia, Oceania	Aboriginal Health Workers (AHWs)	No general cadre description provided	**Screening.**<br><br>Screening for general ear-disease was performed by one AHW who carried out a otoscopic examination of the ear. If the child was determined as needing antibiotic ear drops these were administered by the school nurse.	**Content:** Discussion of the anatomy and physiology of the ear and hearing, showing slides and discussion of the symptoms and treatment of ear conditions, and demonstration of practical skills and ear exam<br>**Duration:** One day<br>**Trainer:** Medical doctor	No details	213 children were screened.<br><br>43% of the aboriginal children had on-going active ear disease vs 7% of white children.<br><br>Microsuction and solfradex were given to the children identified as having ear disease by the school nurse.	No details
Ramesh et al.	Low cost calibrated mechanical noisemaker for hearing screening of neonates in resource constrained settings.	2012	India, South Asia	Accredited Social Health Workers (ASHAs) and Anganwadi Health Workers	No general cadre description provided.	**Screening.**<br><br>Hearing screening of neonates aged between 1 and 30 days was carried out by the health workers under	**Content:** Training on the behavioral response of neonates to noisemakers. Training material was based on the WHO training manuals.	No details	425 neonates of whom 20 had confirmed severe-profound hearing impairment were screened by the ASHA health workers and the anganwadi health workers.	The cost of manufacturing four noisemakers was estimated to be 500 rupees (approximately $8USD).

						the supervision of an audiologist. Mechanical calibrated noisemakers of 50, 60, 70 and 80 dB (A) were used to elicit the behavioural responses. This was validated against a battery of other hearing tests including auditory brain stem response (ABR), otoacoustic emissions (OAE) and behavioural assessment at 2 years of age	**Duration:** 30 hours of training over a period of three months **Trainer:** ENT specialists, audiologists and social workers		The sensitivity and specificity was high with least false positive referrals for 70 and 80 dB (A) noisemakers. All the noisemakers had 100 per cent negative predictive value. 70 and 80 dB (A) noisemakers had high positive likelihood ratios of 19 and 34, respectively	

Ramkumar et al.	Tele-ABR using a satellite connection in a mobile van for newborn hearing testing.	2013	India, South Asia	Village Health Workers (VHWs)	No general cadre description provided	**Screening.**<br><br>Hearing screening was conducted of neonates.<br><br>Auditory Brainstem Response (ABR) testing was conducted in a mobile van with satellite Connectivity by an audiologist, with the help of trained VHWs.<br><br>ABR recordings in newborn babies made by tele-medicine were compared with recordings made face to face.	**Content:** Training on conducting auditory brainstem response<br>**Duration:** No details<br>**Trainer:** No details	Yes. Telemedicine was used. Village health workers and a technician used a van which was equipped with a bed, air-conditioner and a generator power supply. A laptop was used in the van with the auditory evoked response software. There was also a videoconferencing system connected by satellite and the van was controlled for noise.	24 neonates aged between 8 and 30 days were screened.<br><br>The authors found that there was no significant difference between ABR results between the face-to-face method and the telemedicine method.<br><br>As such the authors concluded that real-time tele-ABR testing as a component of newborn hearing screening is feasible in a mobile van using satellite link with the assistance of village health workers.	No details
Ramkumar et al.****	Validation of DPOAE screening	2018	India, South Asia	Village Health	No general cadre	**Screening.**<br><br>Hearing	**Content:** No details	Yes – children referred in the second	One hundred and nineteen children were selected using random	No details

	conducted by village health workers in a rural community with real-time click evoked tele-auditory brainstem response			Workers (VHWs)	description provided	screening of children under the age of five was performed in the child's own home. DPOAE was performed by the VHW.<br><br>Two portable GSI Audioscreener + hand-held devices, which display automatic screening results as ''pass'' or ''refer'', were used for DPOAE screening. As a gold-standard comparision tele-ABR was also used. This took place via tele-medicine. An audiologist in a remote location undertook the	**Duration:** No details<br>**Trainer:** No details	screening underwent tele diagnostic ABR testing in a mobile tele-van using satellite connectivity or at local centre using broadband internet at the rural location.	sampling from 2880 infants and young children under the age of five years<br><br>Tele-ABR and DPOAE findings were compared for 197 ears. The sensitivity of DPOAE screening conducted by the VHW was 75%, and specificity was 91%. The negative and positive predictive values were 98.8% and 27.2%, respectively.<br><br>The authors concluded that the validity of DPOAE screening conducted by trained VHW was acceptable. This study supports the engagement of grass-root workers in community-based hearing health care provision.	

						actual testing, whereas the VHW counseled the child's parents and prepared the child for the ABR testing.				
Ramkumar et al.****	Cost and outcome of a community-based paediatric hearing screening programme in rural India with application of tele-audiology for follow-up diagnostic hearing assessment	2018	India, South Asia	Village Health Workers (VHWs)	VHWs were part of an NGO called the Rural Women's Social Education Centre which focused generally on improving child and maternal health.	**Cost analysis.**<br><br>This study was a cost analysis of the above study.	**Content:** No details<br>**Duration:** No details<br>**Trainer:** No details	Yes – children referred in the second screening underwent tele diagnostic ABR testing in a mobile tele-van using satellite connectivity or at local centre using broadband internet at the rural location.	1335 children under 5 years of age underwent screening by VHWs.<br><br>Nineteen of the 22 children referred completed the tele diagnostic evaluation. Five children were identified with hearing loss. The cost-outcomes were better when using broadband internet for tele-diagnostics. The use of least expensive human resources and equipment yielded the lowest cost per child screened (Rs.1526; $23)<br>When follow-up expenses were maximised, the cost per child was reduced considerably for diagnostic hearing	Yes. A cost-outcome analysis was performed.

									assessment (Rs.102,065; $1532; E1368) and for the cost per child identified (Rs.388,237; $5826)	
Roy et al.	Acute otitis media during the first two years of life in a rural community in Bangladesh : A prospective cohort study.	2007	Bangladesh, South Asia	Community Health Workers (CHWs)	No general cadre description provided	**Screening.**<br><br>This study focused on training CHWs to screen for otitis media amongst children under the age of 2 in rural Bangladesh.<br><br>CHWs were taught how to take a history and observe for signs of AOM. They also took swabs if there was evidence of pus. Where there was no evidence of pus they notified a physician who performed an	**Content:** Training focused on the recognition of acute otitis media. CHWs had specific training on how to take ear swabs. **Duration:** Two weeks of initial training, plus refresher training every two months **Trainer:** Hospital staff at a district hospital	No details	The study was conducted in 10 villages in Mirzapur, a rural subdistrict situated at a distance of 60 km northwest of Dhaka, over a period of 3 years.<br><br>The authors found that forty-six percent (n=115) of the 252 subjects developed AOM: 36% (n=91) during the first year of life and 10% (n=24) during the second year of life (p<0.0001)<br><br>The study documented AOM as an important cause of morbidity among rural children up to two years of age in Bangladesh and concluded that it should be addressed with strategies to overcome the burden of disease.	No details

						otoscopic examination.				
Sánchez et al.	The Potential in Preparing Community Health Workers to Address Hearing Loss.	2017	USA, North America	Community Health Workers (CHWs)	CHWs are employed by the federal government. They have to be members of the community they serve and exhibit leadership characteristics within the context of the community. CHWs then receive on-the-job training, both in the core competencies of CHWs and on specific health conditions	**Training.** This study reported a training program for CHWs to help increase accessibility of hearing health support services in an underserved area.	**Content:** A generalized training course, including identifying signs of hearing loss and appropriate communication strategies. CHWs selected to become community facilitators underwent additional training on liaising with family members **Duration:** 3 hour one day training workshop for all CHWs (n=12) then a 24 hour multisession workshop over 6 weeks for CHWs who would become community facilitators for education and	No details	12 Spanish-speaking local CHWs employed by a federally qualified health center participated in a focus group, twelve received the general training, and four individuals with prior experience as health educators received further in-person training as facilitators of peer-education groups on hearing loss and communication. CHWs increased their knowledge base and confidence in effective communication strategies and developed skills in facilitating hearing education and peer-support groups. They also applied what they had learnt to specific situations, including family support, availability of assistive technology, use of	No details

					such as diabetes.<br><br>They also undertake outreach, advocacy and education, practical instruction, personal assistance (e.g., social support, transportation), and referral services for community members in a language- and culture-appropriate manner.		peer support groups (n=4).<br>**Trainer:**<br>Audiologists and public health researchers		hearing protection, and making referrals for hearing health care.	
Shrestha et al.	Community ear care delivery by community ear assistants and volunteers: a pilot	2001	Nepal, South Asia	Volunteers and Community Ear Assistants (CEAs)	Volunteers in this study were largely women, living in the community. There role	**Screening.**<br><br>This was a community based training program aimed at raising awareness of the burden of	**Content:**<br>The volunteers spent three days learning about the fundamentals of ear disease and care and then the final day	No details	At the time of reporting this community ear care program had been in operation for 18 months.<br><br>The CEA has held 20 primary ear camps and screened 2076 patients,	Mentioned the need for sustainable financing but no details provided on how this was achieved in this particular programme

	programme.				was to inform families about ear disease, inform them about ear screening camps, encourage preventative ear disease measures and refer to the CEA if necessary.<br><br>They were supported by the Community Ear Assistant (CEA) who received more specialist training which has been detailed in the training	ear disease along with prevention.<br><br>The CEA was trained on a variety of issues relating to ear care but specifically in the diagnosis and primary management of acute otitis media.	attending an 'ear camp'.<br>The CAE received general training on ear anatomy, physiology, recognition and management of ear disease, training on communication skills and how to deliver care to communities.<br>**Duration:**<br>The volunteers received four days of training. The CEA received one month of training.<br>**Trainer:**<br>Consultant ENT specialist		with 951 (46%) having AOM.<br><br>Awareness has been raised through stakeholder meetings at the government level about the burden of ear disease.<br><br>There was a 20% increase in the number of patients attending the ENT department at the national hospital which the authors attributed to increased awareness at the community level.	

					column of this table.					
Smith et al.*****	A mobile telemedicine-enabled ear screening service for Indigenous children in Queensland: activity and outcomes in the first three years.	2012	Australia, Oceania	Indigenous Health Workers (IHWs)	No general cadre description provided	**Screening.**<br><br>This was a community based hearing screening service, whereby an IHW screened children in the community using video otoscopy, tympanogram and audiometry. Where necessary the findings were then sent via telemedicine to an ENT specialist for online assessment or children were referred to a local doctor for treatment.	**Content:** No details<br>**Duration:** No details<br>**Trainer:** No details	Yes – telemedicine was used where needed.<br><br>Information was sent to ENT specialists in Brisbane for online review.<br><br>Of the 2111 assessments performed during this period, 562 referrals (27%) were made to an ENT specialist for online review.<br><br>More than half of all cases reviewed by the specialist were nominated for review (34%) or surgery (21%) at the	During the first three years of the programme a total of 2111 screening assessments were carried out at 21 schools in the remote Queensland region.<br><br>The average screening rate achieved in the community was 85%. More than half of all assessments resulted in a referral to the ENT specialist (for online assessment) or local doctor (for treatment). Twenty specialist ENT online clinics were conducted during which 415 patients were reviewed. Over half of all online review cases (55%) resulted in appointments at the next ENT outreach clinic for further review and/or surgery.	No details

								next ENT outreach clinic in Cherbourg. During the three year period, there were six cases identified which needed more urgent treatment in Brisbane.		
Smith et al.*****	Monitoring ear health through a telemedicine-supported health screening service in Queensland	2015	Australia, Oceania	Indigenous Health Workers (IHWs)	No general cadre description provided	**Screening.**<br><br>This was a three-year follow-up study regarding the above hearing screening program.	**Content:** No details<br>**Duration:** No details<br>**Trainer:** No details	Yes – see above description from 2012 Smith study.	The authors reported on the total number of children screened (n=5539) since the establishment of the screening programme in 2009.<br><br>Screening activity has increased by more than 50% since 2009, there has been a slight reduction in the proportion of children failing assessment, with the mean failure rate changing from 33% in 2009 to 26% in 2014.	No details
Wagner et al.*	Monitoring of hearing and	2017	Brazil, South America	Community Health	CHAs are considered to be the	**Screening.**	**Content:** Risk factors for hearing	No details	Of the 41 CHAs invited to take part in the hearing screening program, two	No details

	language in primary health care: project pilot.			Agents (CHAs)	link between the community and the Family Health Strategy (FHS) team. They typically have a role in promotion and disease prevention activities and in educational and monitoring actions for individuals, families and groups.	This study reports on a community based neonatal hearing screening program carried out in the community by CHAs.<br><br>This study used the questionnaire validated by Alveranga in the 2012 study.<br><br>The questionnaire was used with children's parents and caregivers to try and identify early onset hearing loss in newborns on a monthly basis.	impairment, prevention, classification and rehabilitation of hearing losses<br>**Duration:** 4-hours<br>**Trainer:** University staff		refused to take part, five gave up participating. Twenty-six (66.66%) did not perform monitoring, seven (17.94%) mon-itored improperly and only six (15.38%) moni-tored properly.<br><br>Only one child was identified at being at risk of hearing loss from the questionnaire based screening as was referred to the hospital for further assessment.<br><br>In its current format this form of screening was deemed unsatisfactory. More attention should be paid to the burdens placed on CHAs and different forms of screening identified through working in partnership with CHAs.	
William s et al.	Basic audiologica l and related	1980	Canada, North America	Native Technicia ns	The native technician was a local from the	**Treatment.**<br><br>This was a descriptive	**Content:** Hearing testing, anatomy and physiology of the	No details	10 hearing aids were fitted and fixed by the hearing technician. This is really important as prior	Private philanthropy fund funded the salary of the technician

	services in the north -- the role of a native technician.				community with a knowledge of cultural background of the people they served. They were fluent in the local dialects and their main focus was on providing ear and hearing care services.	study outlining the role of a Native Technician in rural Canada to deliver ear and hearing care. The study outlined the training and preliminary results over an 18-month period of the program being in operation.	ear, hearing aid mold taking, trouble shooting, record keeping, introduction to audiometry. Also raising awareness about noise induced hearing loss **Duration:** Mentioned 'several weeks of training and on-going supervision' including three weeks in a hospital setting doing supervised examination, auditing a course for audiologists, and one week with the hearing aid distributor **Trainer:** Multiple trainers – see above.		to this all hearing aid issues could only be fixed by taking the hearing aids and flying them to a major city - this was a problem as it didn't matter how minor or major the defect was the solution was always this. Often the hearing aids were lost which caused great inconvenience to the wearer. The paper comments that this is no longer a problem. The authors also comment about raised awareness amongst community members about noise induced hearing loss.<br><br>They conclude that this program could compliment the work of ENT surgeons in the area, especially in geographically remote areas where trips to major centres are often expensive, and lengthy both in terms of distance and time.	and year-by-year contractual funding was found for the audiologist but no figures provided or formal cost analysis done.
Youngs et al.	Diagnostic otoscopy skills of	2011	Nepal, South Asia	Community Ear	CEAs work as part of a service	**Training.**	**Content:** Training was on	No details	The CEAs examined the ears of 92 potential surgical patients and also	No details

	community ear assistants in Western Nepal.			Assistants (CEAs)	delivered by the Britain Nepal Otology Service, in association with the Nepal Red Cross. CEAs are workers with basic technical qualifications who receive intensive training in the prevention, diagnosis and treatment of ear disease.	This report provided a general overview of the role CEAs have, while specificially assessing their diagnostic otoscopy skills.	diagnostic otoscopy skills. **Duration:** No details **Trainer:** No details		viewed 50 images on a computer screen. They were expected to pick up any pathology. This was then compared with consultant ENT surgeons and junior doctors diagnoses.<br><br>Overall the selection of patients for middle-ear surgery concurred with the consultant otologists' opinion in 87 of 92 patients (94.5 per cent). The level of community ear assistants' otoscopy skills was between that of junior and senior otolaryngology trainees.<br><br>There were no cases in which the community ear assistants had failed to diagnose significant otological pathology.	

Supplementary Table 4. Information from final studies. A table outlining key details from studies included in the final review.

Key:

* Linked studies. The 2017 study by Wagner et al., utilized the screening questionnaire to try and identify hearing loss in children under the age of one, which was originally reported on in the study by Alveranga et al., in 2013.

** Linked studies. The 2005 study by Couzos et al. was an in-depth report outlining the issues and principles that underpin community-controlled health research which formed the basis of the 2003 Couzos et al. study in which the role of Aboriginal Health Workers to identify and treat CSOM in the community was assessed.

*** Linked studies. The 2008 study by Olusanya et al. detailed a hearing-screening program for neonates that utilized oto-acoustic emissions and automated auditory brainstem responses to screen neonates and children for hearing loss. The 2009 study by the same authors was in-depth analysis of the 2008 study, aimed at understanding factors associated with follow up.

**** Linked studies. The 2018 studies by Ramkumar et al. reported on a hearing-screening program for children under the age of five was performed by a VHW. One study outlined results from the screening programme, the other reported a cost-utility analysis of the study.

*****Linked studies. The 2012 study by Smith et al., outlined a community ear program lead by Indigenous Health Workers, whereby children were screened in the community using video otoscopy, tympanogram and audiometry. Where necessary images were sent via tele-link to an ENT specialist for review and follow up. The 2015 study by the same authors was a 3-year follow up report.
